# Kininogen Cleavage Assay: Diagnostic Assistance for Kinin-Mediated Angioedema Conditions

**DOI:** 10.1371/journal.pone.0163958

**Published:** 2016-09-29

**Authors:** Rémi Baroso, Pauline Sellier, Federica Defendi, Delphine Charignon, Arije Ghannam, Mohammed Habib, Christian Drouet, Bertrand Favier

**Affiliations:** 1 GREPI EA7408, Université Grenoble Alpes, Grenoble, France; 2 KininX SAS, Grenoble, France; 3 National Reference Center for Angioedema Grenoble, France; 4 Qalam-Antibody, La Tronche, France; University of Thessaly Faculty of Medicine, GREECE

## Abstract

**Background:**

Angioedema without wheals (AE) is a symptom characterised by localised episodes of oedema presumably caused by kinin release from kininogen cleavage. It can result from a hereditary deficiency in C1 Inhibitor (C1Inh), but it can present with normal level of C1Inh. These forms are typically difficult to diagnose although enhanced kinin production is suspected or demonstrated in some cases.

**Objectives:**

We wanted to investigate bradykinin overproduction in all AE condition with normal C1Inh, excluding cases with enhanced kinin catabolism, and to propose this parameter as a disease biomarker.

**Methods:**

We retrospectively investigated high molecular weight kininogen (HK) cleavage pattern, using gel electrophoresis and immunorevelation. Plasma samples were drawn using the same standardised procedure from blood donors or AE patients with normal C1Inh conditions, normal kinin catabolism, and without prophylaxis.

**Results:**

Circulating native HK plasma concentrations were similar in the healthy men (interquartile range: 98–175μg/mL, n = 51) and in healthy women (90–176μg/mL, n = 74), while HK cleavage was lower (*p*<0.001) in men (0–5%) than women (3–9%). Patients exhibited lower native HK concentration (*p*<10^−4^; 21–117μg/mL, n = 31 for men; 0–84μg/mL, n = 41 for women) and higher HK cleavage (*p*<10^−4^; 10–30% and 14–89%, respectively) than healthy donors. Pathological thresholds were set at: <72μg/mL native HK, >14.4% HK cleavage for men; <38μg/mL; native HK, >33.0% HK cleavage for women, with >98% specificity achieved for all parameters. In plasma from patients undergoing recovery two months after oestrogen/progestin combination withdrawal (n = 13) or two weeks after AE attack (n = 2), HK cleavage was not fully restored, suggesting its use as a post-attack assay.

**Conclusion:**

As a diagnostic tool, HK cleavage can offer physicians supportive arguments for kinin production in suspected AE cases and improve patient follow-up in clinical trials or prophylactic management.

## Introduction

Angioedema manifests as self-limiting oedema affecting the subcutaneous and sub-mucosal tissues. It is most commonly associated with wheals in conjunction with urticaria, an histamine mediated-, mast cell degranulation related disease [1,2]. Angioedema without wheals (AE) is considered an independent symptom [1], and has been defined as nonpruritic, nonpitting, and resistant to H1 antihistamine drugs [3,4]. AE can prove life-threatening if the upper airways are affected, though symptom intensity and frequency vary widely, even within the same family [3,4]. AE is usually divided according to its hereditary or acquired origin [1], but other classifications have been proposed [2,5] and we will use the level of functional C1Inhibitor (C1Inh) as the main classification factor.

AE with C1Inh deficiency (C1Inh-AE) can be acquired or linked to mutations in the *SERPING1* gene (OMIM 106100, estimated prevalence: 1/50,000), which is the best characterised form of AE [6]. Bradykinin (BK) is recognized as the predominant mediator in C1Inh-AE [7,8]. In contrast, the pathogenesis of AE with normal C1Inh level (nC1Inh-AE) remains incompletely understood, though response to BK receptor antagonist indicates that BK may also be involved. In Factor XII-related AE (FXII-AE), causative mutations have been identified in the Factor XII encoding gene (OMIM 610618) [1,9–11], leading to pathological BK production [12]. Yet the mutation alone cannot explain the diversity of the disease among the same family and FXII-AE is multifactorial [13,14]. The nC1Inh-AE can also occur without FXII mutation, triggered by oral oestrogen/progestin combination (OP) intake [15–17] through possible transcriptional regulations which remain to be formally explicated [5]. Since BK is degraded by several peptidases, including angiotensin-converting enzyme (ACE), ACE inhibitor treatment can trigger AE (ACEi-AE, OMIM 300909) [1,5,18] but other BK degrading peptidases might also be implicated [2,18]. Finally there are idiopathic or even allergic AE (I-AE), where the relative involvement of BK and histamine pathways is unclear and noticed mainly by response to treatment [1,5,19].

BK is produced from the cleavage of high-molecular weight kininogen (HK) by kallikrein (KK) upon contact-system activation [20]. KK circulates in the plasma as prekallikrein, a zymogen complexed to HK [21], which can be activated by Factor XII protease (FXIIa) within a positive feedback loop [22]. Contact-system activation is controlled by C1Inh, which inhibits FXIIa and KK activities *via* serpin-protease association [23]. Plasma kinin concentration is not pertinent to kinin-AE diagnosis due to the short half-life of BK and its active metabolite *des*Arg^9^-BK [24] (27±10s and 643±436s, respectively). Biological diagnosis of C1Inh-AE is therefore based on reduced C1Inh function, using C1s [1] or contact-phase protease [25] as target, while hereditary C1Ihn-AE is also diagnosed on detection of *SERPING1* mutations. Diagnosis of nC1Inh-AE is still controversial: detection of *F12* gene mutation is pertinent only to FXII-AE but cannot explain why many carriers are asymptomatic [13,14]. Enzymatic tests have been performed to investigate increased kinin-forming conditions [26], C1Inh-protease complexes [27] or defective kinin catabolism [13,28]. While some of these tests are already used in AE classification [2], their consensual use is still pending on pre-analytical and enzymatic considerations [29].

In contrast to BK, HK has a long life in bloodstream, with a 3-day regeneration time [30]. HK is a 120kDa α-globulin protein composed of six domains, the fourth of which generates BK [31]. HK cleavage can adequately reflect BK release, indicating contact phase activation, under appropriate pre-analytical standardisation. This prompted us to investigate plasma HK in established AE conditions and to set up an assay with consistent arguments for BK production.

## Material and Methods

### Reagents

Purified native (single chain) HK (N-HK) and horseradish-peroxidase-conjugated anti-HK light chain polyclonal antibody were purchased from Enzyme Research (Swansea, UK). HK purity was estimated at >99% by the manufacturer. We confirmed a batch purity >90% by means of silver staining a poly-acrylamide gel under reducing conditions and calculated the concentration using bicinchoninic acid assay (microBCA, Interchim, Montluçon, France) and optical absorption, taking *E*1%/cm at 280mm = 7.01, according to the manufacturer’s instructions. Other chemicals were purchased from Sigma (Saint Quentin Fallavier, France).

### Patient samples

Control plasma samples were drawn from blood donors, including healthy males (n = 51, including samples H1–H5), females (n = 74) including some without oral oestrogen/progestin (OP) contraception (n = 30, H6–H10) and some on OP contraception (n = 32). AE diagnosis was established by the physicians of the French Reference Centre for Angioedema (CREAK) network according to the criteria of kinin-AE clinical presentation [4]. Samples from patients under treatment (tranexamic acid, danazol, or C1Inh) were excluded from participation. Given the uncertainty of their diagnosis, I-AE cases were not included. In order to eliminate potential interference from enhanced kinin half-life, we excluded patients with detectable defects in kinin catabolism from the main study and kept three samples for comparison (K1–K3). Patient samples were further categorised according to C1Inh function and genetic data. We analysed four C1Inh-AE samples (C1–C4). The main study included all remaining nC1Inh-AE patient samples (n = 31, men, M1–M9; n = 41, women, W1–W6). We also examined HK in plasma samples from an already published cohort of nC1inh-AE women taking OP contraception (n = 13, O1–O6) [32].

### Gel electrophoresis and HK quantification

We submitted 0.5μL of plasma to 8% poly-acrylamide gel electrophoresis with sodium dodecyl sulphate (SDS-PAGE) under reducing conditions (2-mercapthoethanol, 2.5%), followed by transfer onto nitrocellulose membrane (Hybond^™^, GE HealthCare, Chalfont St. Giles, UK), saturation with 5% non-fat dry milk in TBST buffer (150mM NaCl, 10mM Tris, pH 7.5, 0.01% Tween 20), and incubation with a horseradish peroxidase-conjugated anti-HK light chain polyclonal antibody at 1/25,000 dilution in TBST buffer. Using enhanced chemiluminescence (Clarity Western ECL^™^ substrate, Biorad, Hercules, USA) and densitometry scan quantification (ChemiDoc^™^ XRS+ System camera and Image Lab^™^ software, Bio-Rad, Hercules, USA), three molecular species were retained for data processing: native protein (N-HK, 120kDa), light chain (LC-HK, 56kDa), and cleaved light chain (cLC-HK, 46kDa). A linear regression was plotted using HK standard and densitometry. N-HK concentration in plasma was calculated by dividing the estimated mass on the gel by the loaded sample volume (0.5μL). Precision (gel load and transfer) was monitored by the correlation coefficient between the HK standard protein load onto the gel and densitometric signal. The HK cleavage percentage was defined as the ratio of both light chain signal to the sum of the three bands: (LC-HK + cLC-HK) / (N-HK + LC-HK + cLC-HK).

### HK standard and plasma sample preparation

Purified N-HK was mixed with loading buffer containing SDS (2%, w/v) and β-mercaptoethanol (2.5%, v/v), then boiled and stored frozen. One aliquot was thawed daily, then further diluted in loading buffer prior to loading known amounts on gels (typically 20, 40 80 and 110 ng).

Human blood was drawn in plastic citrated tubes and shipped at 20–25°C within two days. Platelet-free plasma was collected following centrifugation at 2,000×g for 10min at 20°C, then aliquoted avoiding any platelet contamination, and frozen at -80°C until use. These standardised preparations were performed in the same laboratory for all samples, by trained technicians, to ensure reproducibility and minimal contact phase activation, as already described [2,13,16,25,26,33]

### C1 Inhibitor function, kinin forming, and catabolism activities

C1Inh function was measured using C1s protease as target [34]. Kinin-forming activity was assessed by measuring plasma spontaneous amidase activity using the chromogenic peptide HD-Pro-Phe-Arg-*p*NA [26]. Kinin catabolism was indirectly investigated by measuring aminopeptidase P, carboxypeptidase N, and angiotensin-I converting enzyme (ACE) activities [13,28].

### Statistics

Statistical analysis was performed using GraphPad Prism^®^ 6 software (GraphPad Inc, La Jolla, USA). All results were expressed as median {quartile 1-quartile 3} and compared using the non-parametric Mann-Whitney-Wilcoxon test. Box-and-whisker plots were drawn with whiskers extending to 5% and 95% values, and the corresponding numerical values were reported in the figures in addition to the quartile 1, median, and quartile 3. Paired samples were compared using the Wilcoxon matched-pairs signed rank test. Cut-off values were determined in order to maximize the sum of sensitivity plus specificity using receiver operating characteristic (ROC) curves [35]. Positive and negative predictive values were calculated using the samples of this study, not the estimated prevalence of AE.

### Ethics

All procedures were performed according to the principles of the Helsinki declaration and French ethical policies governing the use of biological sample collection (Ministry of Health authorization DC-2008-634). All patients and blood donors provided written informed consent to participating in the investigation. Grenoble university hospital’s institutional review board (IRB South-East committee V) specifically approved this study. All data was anonymously processed.

## Results

### Human plasma kininogen concentration differs between men and women

**Fig 1:**
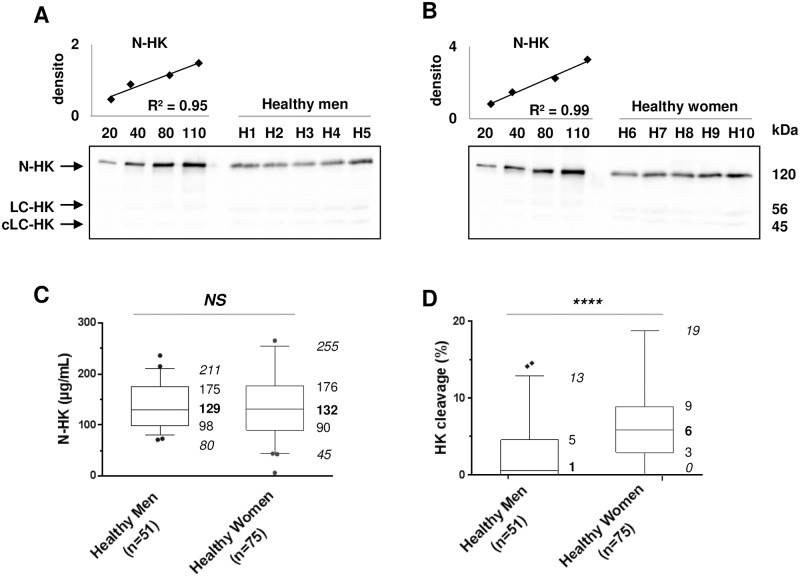
Quantification of HK molecular pattern in control male and female samples. **A, B**: Purified human HK (20, 40, 80, and 110ng) and 0.5μL of plasma from five male healthy donors, (H1 to H5) (A), and five females (H6 to H10) (B) were subjected to SDS-PAGE. The signal of HK native chain (N-HK), light chain (LC-HK), and cleaved light chain (cLC-HK) were quantified by densitometry and N-HK concentration was evaluated by the displayed linear regression. **C, D**: Box-plot displaying 5–95% range (*italic* fonts), median (**bold** fonts), and interquartile values of plasma concentration of N-HK (C) and HK cleavage (D), measured as in panel A. densito: densitometry in 10^7^ arbitrary units; NS: non-significant; ****: *p*<10^−4^ (Mann-Whitney-Wilcoxon test).

The BK release depends on cleavage of native HK (N-HK) by KK into heavy (64kDa) and light (56kDa, LC) chains. LC-HK is further cleaved into a cleaved light chain (46kDa, cLC) [36,37]. N-HK concentration and HK cleavage were investigated in plasma by means of immunoblotting. Purified single-chain HK protein was used as standard to evaluate N-HK plasma concentration in healthy blood donors. Precision of the gel load and transfer was controlled using the linearity between the protein mass loaded on the gel and the densitometric signal (Fig 1A and 1B). The median N-HK concentrations and {interquartile range} were 129 {98–175} μg/mL for men (n = 51) and 132 {90–176} μg/mL for women (n = 75, Fig 1C). The 56- and 45-kDa bands, corresponding to LC-HK and cLC-HK, respectively, were quantified to evaluate HK cleavage, as described in the Material and Methods section. The healthy plasma HK cleavage revealed a statistical difference (p<10^−4^) between men (1% {0–5}) and women (6% {3–9}) (Fig 1D). Time delays between AE patient blood sampling and processing, and plasma freeze-thaw cycles were analysed (n = 3). Neither 2-day room temperature delay of whole blood storage nor five freeze-thaw cycles of plasma modified the distribution of HK molecular species (data not shown).

### Kininogen cleavage for angioedema diagnosis

We performed HK evaluation in C1Inh-AE patients (n = 9), with four samples displayed in S1 Fig. N-HK was not detectable in patients C1, C2, or C3, whereas very low amounts were detected in C4. HK cleavage was almost complete in these patient samples, in line with high kinin-forming activity, as previously reported [26,38].

**Fig 2:**
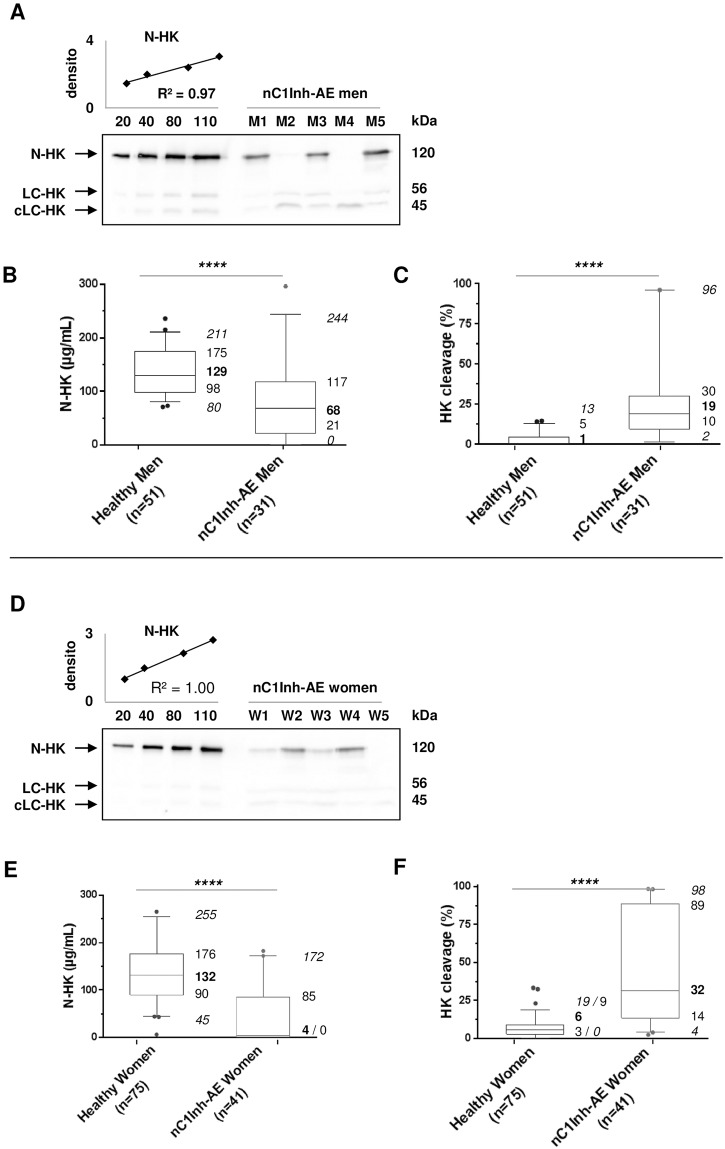
N-HK concentration and HK cleavage in nC1Inh-AE patient samples. **A**: Purified human HK (20, 40, 80, and 110ng) and 0.5μL of plasma from nC1Inh-AE male patients (M1 to M5) were subjected to SDS-PAGE. The signal of HK native chain (N-HK), light chain (LC-HK), and cleaved light chain (cLC-HK) were quantified by densitometry and N-HK concentration by the displayed linear regression. **B, C**: Box-plot displaying 5–95% range (*italic* fonts), median (**bold** fonts), and interquartile values of plasma concentration of N-HK (B) and HK cleavage (C), measured as in panel A, compared to the healthy cohort studied in Fig 1. **D, E, F**: as in A, B, C, concerning female patients (W1 to W5). densito: densitometry in 10^7^ arbitrary units; ****: *p*<10^−4^ (Mann-Whitney-Wilcoxon test); nC1Inh-AE: angioedema with normal C1 inhibitor.

We then analysed HK patterns in nC1Inh-AE patients. The median N-HK concentrations were 68μg/mL {21–117} for men (n = 31) and 4μg/mL {0–85} for women (n = 41). The median HK cleavage was 19% {10–30} for men and 32% {14–89} for women (Fig 2). N-HK concentration and HK cleavage significantly differed between nC1Inh-AE patients and healthy donors (*p*<10^−4^), suggesting that both measurements could be used as AE diagnostic tools.

HK cleavage calculation can be expressed as (total HK—N-HK) / (total HK), which anticipates a linear relationship between N-HK and HK cleavage if total HK is stable. The correlation value between N-HK concentration and HK cleavage was low (r^2^ = 0.38 for men, r^2^ = 0.56 for women, S2A and S2B Fig), suggesting that the overall HK concentration (N- + LC- + cLC-HK) was discontinuous during the *in vivo* HK cleavage process, and that the light chain would be rapidly degraded in plasma. We confirmed in healthy plasma (n = 3) that total HK did not remain constant upon dextran sulphate activation: N-HK almost disappeared, while LC-HK and cLC-HK only slightly increased (data not shown). Investigating N-HK concentration and HK cleavage together thus appears appropriate for analysing HK molecular species in patient plasma. Both parameters were plotted against the kinin-forming activity of the samples assayed as described [26] as indicators of active HK-specific proteases (data not shown). The correlation was not complete, suggesting that N-HK concentration and HK cleavage provided data that was not completely redundant with that of kinin forming activity.

**Fig 3:**
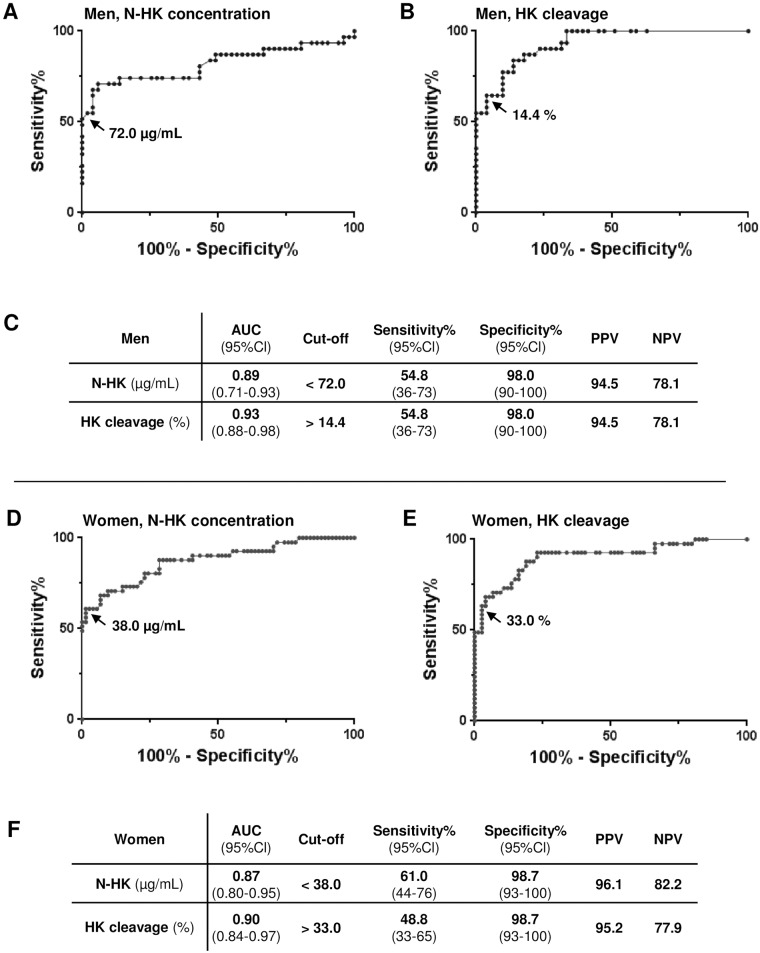
Threshold of N-HK concentration and HK cleavage. **A, B**: ROC curve analysis between healthy men and nC1Inh-AE male patients of N-HK concentration (A) and HK molecular species (B), displayed in Fig 2B and 2C, respectively. The arrows point to the dots corresponding to the maximum of the sum {sensitivity + specificity} and the corresponding thresholds. **C**: Figures associated with the ROC curve analysis shown in A and B along with their calculated 95% confidence levels (95% CI). **D, E, F**: as in A, B, C for indicators on women displayed in Fig 2E and 2F. AUC: area under the curve; PPV: predictive positive value; NPV: negative predictive value; N-HK: native single-chain of HK.

ROC analysis was performed as described [35] for both parameters to determine the thresholds offering maximum sum of specificity plus sensitivity (Fig 3). For the men, the optimal cut-off values were <72.0μg/mL for N-HK and >14.4% for HK cleavage (98% specificity; >94% PPV). The optimal cut-off values for the women were <38.0μg/mL for N-HK and >33.0% for HK cleavage (>98% specificity, >95% PPV). The indicators of performance for AE diagnosis were excellent for both specificity and PPV.

We next investigated whether experimental conditions and technical skill would influence the results in the event of an extended diagnostic tool application. We estimated the inter-assay variability at 34% for N-HK concentration (3 healthy plasma, tested n = 5, 5 and 3 times respectively, S1 File). Given the low signals achieved with LC-HK and cLC-HK bands, we modified the immunoblotting procedure described in the Material and Methods section to maximize the quantification of faint bands. N-HK concentrations were found to change when using both protocols (correlation R^2^<0.6, S2C and S2D Fig), yet correlation between HK cleavage data remained high (R^2^>0.8), with a slope close to the unity, and a mean deviation between both protocols of 10% (S1 File). We concluded that estimation of HK cleavage is less susceptible to experimental conditions than N-HK concentration measurement, and would therefore anticipate good reproducibility between laboratories.

### Applications for angioedema situations

**Fig 4:**
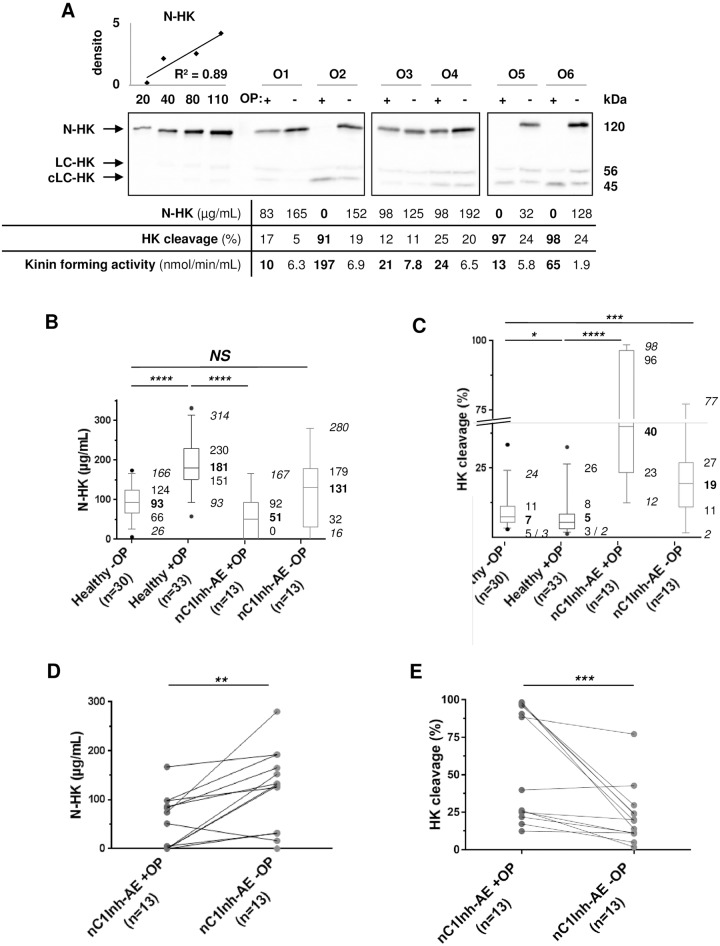
HK concentration in samples from nC1Inh-AE patients taking oral contraception. High-molecular-weight kininogen (HK) molecular pattern of plasma samples from healthy women taking or not estrogen/progestin combination (OP; Healthy+OP, n = 32; Healthy-OP, n = 30) and from nC1Inh-AE patients taking OP and ≥2 months after OP withdrawal (nC1INh-AE+/-OP, n = 13). **A**: Analysis of plasma samples (0.5μL/lane) from six patients (O1–O6) and the corresponding figures. N-HK concentration of P1 and P2 was evaluated by the displayed linear regression, kinin-forming activity was determined as in [26], pathological values were labelled in **bold** fonts. **B, C**: Box-plot displaying 5–95% range (*italic* fonts), median (**bold** fonts), and interquartile values of the plasma concentration of N-HK (B) and cleaved HK species (C), measured as in panel A. **D, E**: Details of the paired samples of panel B and C respectively analysed using the Wilcoxon matched-pairs signed rank test. densito: densitometry in 10^7^ arbitrary units; *NS*: non-significant; *: *p*<0.05; **: *p*<0.01; ***: *p*<0.001; ****: *p*<10^−4^ (non-parametric tests); N-HK, LC-HK, cLC-HK: native chain, light chain, cleaved light chain of HK respectively; nC1Inh-AE: angioedema with normal C1 inhibitor.

Several precipitating factors for AE clinical phenotype have already been described, *e*.*g*. OP contraceptive intake [15–17]. We recently reported a series of 18 women suffering from nC1InhAE attacks while taking OP contraception and undergoing symptom remission after pill withdrawal [32]. As an example of this pathological condition, 13 samples from this cohort were investigated and compared to healthy women under OP. N-HK concentration was decreased during OP intake and restored after hormone withdrawal (n = 6; Fig 4A, P1–P6). These healthy women displayed higher N-HK concentration (p<10^−4^) and HK cleavage levels somewhat lower (p<0.05) to those of the healthy cohort whose contraception methods do not include OP (Fig 4B and 4C). For the 13 patients under OP contraception (nC1Inh-AE+OP), N-HK plasma concentrations were significantly lower compared to controls (51μg/mL {0–92}; Fig 4B; *p*<10^−4^), and HK cleavage was higher (40% {23–96}; Fig 4C; *p*<10^−4^). After OP withdrawal (nC1Inh-AE-OP), N-HK plasma concentrations increased (131μg/mL {32–179}; Fig 4D; *p*<0.1) close to the values expected from healthy women without OP (Fig 4B; NS). In contrast HK cleavage after OP withdrawal was attenuated (19% {11–33}; Fig 4E; *p*<0.001) for most women, but remained more abundant compared to controls (Fig 4C; *p*<0.001), with some samples exhibiting values within the range usually found in nC1Inh-AE conditions. This demonstrated that most of these patients did not fully recover the normal conditions observed in healthy women. In this example, HK cleavage analysis provided pertinent information on the patients’ kinin production, more so than kinin-forming activity measurement.

**Fig 5:**
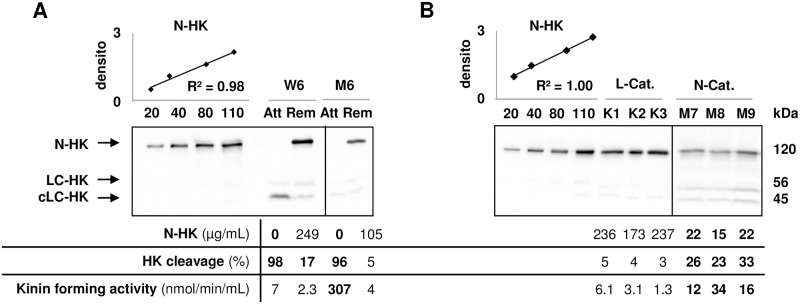
HK molecular pattern analysis of AE patients. **A**: High-molecular-weight kininogen (HK) molecular pattern of plasma samples (0.5μL/lane) from two nC1Inh-AE patients (W6, M6) less than 24h after the onset of an AE attack (Att) and at least two weeks after remission (Rem), analysed as in Fig 1. **B**: Same as for A, with plasma samples from three patients (K1–K3) with ACEi-AE and low kinin catabolism (L-Cat) and three patients (M7–M9) with nC1Inh-AE and normal kinin catabolism (N-Cat). N-HK concentrations were evaluated by the displayed linear regressions, kinin-forming activity was determined as in [26], pathological values were labelled in **bold** font. densito: densitometry in 10^7^ arbitrary units; N-HK, LC-HK, cLC-HK: native chain, light chain, cleaved light chain of HK respectively.

To further evaluate the usefulness of HK cleavage in patient follow-up or clinical investigation, we compared plasma samples drawn during AE attack (*i*.*e*. within 24h after its beginning) with samples drawn in remission period, more than two weeks after the symptom relief (Fig 5A). All samples were shipped and processed within 48h as described in the Material and Methods section. N-HK species were not detectable in samples taken during attacks. During remission, N-HK concentrations were restored to normal values, as were the kinin-forming activities, though W6 patient still displayed an HK cleavage value slightly above threshold.

AE can also be attributed to ACE-inhibitor (ACEi) intake, probably through a lower kinin catabolism and accumulation of BK on its receptors [39]. We therefore asked the question of whether ACEi administration would affect HK cleavage. N-HK concentration and HK cleavage were thus evaluated in ACEi-AE patients (n = 3; Fig 5B, K1–K3) and compared to nC1Inh-AE patients with normal kinin catabolism (Fig 5B; M7–M9). In contrast, N-HK concentration, HK cleavage and kinin-forming activities were within normal ranges for all ACEi-AE plasma. This suggests the aetiopathology of ACEi-AE is not associated with enhanced HK proteolysis and subsequent kinin overproduction. This underlines the interest of our unambiguous test for BK overproduction to decipher the pathological mechanisms of AE.

## Discussion

The present work develops biological arguments for the involvement of kinin in AE symptoms based on observations of plasma samples. It has been established that BK and its metabolite *des*Arg^9^-BK display very short half-lives in plasma (27±10s and 643±436s, respectively) [24]. In contrast, HK exhibited a long (3 days) half-regeneration time [30] and its cleavage kinetics were fast [36,37]. HK cleavage can thus be considered a direct indicator of BK release and therefore a pertinent indicator of kinin-mediated AE [40]. Our assay was based on HK molecular species analysis in samples, presenting variable distribution from almost entirely native HK (indicating a steady state with minimal and physiological BK production) to the presence of a few remaining cleaved light chains (indicating maximal BK production). The reconstitution of plasma native HK by liver production is slow, so the observed distribution deals with a robust readout of the *in vivo* kinin production during the active period of AE.

Patients with detectable kinin catabolism defects could also be at risk of AE symptoms due to kinin receptor over activation [2]. This condition is consistent with a local pathogenic process, but does not claim a systemic activation associated with HK cleavage [29]. These patients have not been included in the nC1Inh-AE cohort we investigated here in order to avoid false-negative data from individuals presenting with an AE phenotype and no HK cleavage. This coherent exclusion biased the specificity of our assay; the three patients presented in Fig 5B were an indirect example that nC1Inh-AE can include two different (non-exclusive) mechanisms: higher BK production (a context of our main cohort) and lower catabolism, as recently reported by Dessart *et al*. [2]. The present assay will provide an additional contribution to support this distinction.

Concerning female individuals, our results in Fig 4 are congruent with contact-phase activation and subsequent HK cleavage upon oestrogen trigger [16]. However, some of our example female patients did not fully recover from low HK cleavage (Fig 4; W6 in Fig 5), while N-HK and kinin-forming activity returned to normal values. Compared to control individuals, this remnant HK cleavage might indicate susceptibility to AE attacks, promoting HK cleavage as a key parameter when deciding whether to administer prophylactic treatment of AE.

While several studies have already demonstrated cleavage of single-chain HK in AE [26,38,40], this report was the first to quantify the phenomenon while using two parameters to support the diagnostic tools in routine application. Of these two parameters, HK cleavage was the least susceptible to experimental conditions (S2 Fig), a key feature to compare in future studies. In contrast, N-HK concentration values were influenced by experimental conditions. This could partially account for the discrepancy between the N-HK concentrations reported here and the 70–90μg/mL or 69–116μg/mL ranges of total plasma HK reported in previous studies ([41] and [42], respectively). The amount of cleaved HK proteins (in healthy plasma or, probably, in the standard used) might also partly explain the differences. The salient point remains, however, that N-HK concentration figures must be interpreted separately in the context of each experimental protocol.

In this report, we observed a highly significant difference in N-HK concentration values (*p*<0.001) between healthy women taking OP contraception and those without this hormonal treatment. This is consistent with the possible transcriptional regulation of the some contact phase proteins by sexual hormones (exemplified by the efficiency of androgen in AE prophylaxis, reviewed in [5]), and the relevance of oestrogen intake as AE pathological trigger ([32], (Fig 4).

We checked that our pre-analytical procedure did not induce *ex vivo* contact phase activation of healthy plasma. We assumed one single freeze/thaw cycle not to worsen HK cleavage in the context of low C1Inh control. However, the opposite situation would not impair HK cleavage analysis since we strictly applied the same procedure to all samples. Importantly, the use of EDTA, heparin or polybrene, although compatible with HK analysis [43], would not absolutely protect HK from cleavage in extensive proteolytic conditions [44] but would preclude other enzymatic analysis [25,26].

This HK investigation can serve alongside the already-established C1Inh assay and kinin-forming activity measurement to provide the physician with a positive identification supporting a kinin-AE condition. While C1Inh assays have little relevance in the context of nC1inh-AE, kinin forming activity assay is theoretically limited by the specificity of the substrate used for plasmatic KK. The option to analyse HK cleavage is appropriate for difficult diagnoses when patients present with an uncommon clinical phenotype, *e*.*g*. nC1Inh-AE, or an ambiguous one, *e*.*g*. with participation of the mast cell component. A primary advantage of this complementary assay lies in its high specificity, rather than sensitivity [45], which prompts us to propose this method as a positive criterion of a kinin-dependent AE condition.

Its lower sensitivity can probably be explained by the data displayed in Fig 5A and 5B. Since sampling delay was not fully determined in terms of the time of attack onset, some samples included in our study (illustrative examples: M5 in Fig 2A, W4 in Fig 2D) may have come from patients having fully or partially recovered from a recent AE attack, thus producing false-negative results. Appropriate, time-controlled sample capture would ensure more precise information is gathered on circulating HK species from initiation to the recovery phase of an AE attack. Another aim of such sampling could be to investigate the relative kinetics of N-HK concentration, HK cleavage, and kinin-forming activity, which have not been numerically correlated, despite offering approximately the same specificity. This might provide insight into contact-phase activation and recovery, together with possible therapeutic implications. However, the fact that AE patients can display above normal HK cleavage in remission period, or almost complete HK cleavage in attack situation, indicates that the local effect of BK release (AE is a *localised* swelling) has to be distinguished from the causal or concomitant plasma protein perturbations (*e*.*g*. plasmatic HK cleavage), as already discussed by Hofman [29].

In conclusion, we performed a semi-quantitative HK assay in order to estimate N-HK concentration and HK cleavage in plasma samples from nC1Inh-AE patients with normal kinin catabolism enzymes. Our assay detected nC1Inh-AE patients with a high specificity, and confirms data from a recent study which was limited to FXII-AE plasma [12]. Our study is a first step toward: (i) demonstrating the role of contact phase in all nC1Inh-AE, or at least to define which angioedema symptoms are BK related, (ii) deciphering the BK pathway in I-AE, and (iii) use of HK cleavage measurement in the follow-up of patients or therapeutic options. Our assay is also anticipated to find applications in injury cases where contact-phase activation is emerging, *e*.*g*. Transfusion-Related Acute Lung Injury (TRALI) and other adverse blood product reactions, with the aims of suggesting adequate therapeutic options [46].

## Supporting Information

S1 Fig Evidence of HK cleavage in C1Inh-AE patients. High-molecular-weight kininogen (HK) molecular pattern of plasma samples from one control (H11) and C1Inh-AE patients (n = 4; C1–C4), analysed as in Fig 1. N-HK concentrations were evaluated by the displayed linear regression, kinin-forming activity as in [26], and pathological values were labelled in bold fonts. densito: densitometry in 10^7^ arbitrary units; C1Inh-AE; angioedema with C1Inh deficiency; N-HK, LC-HK, cLC-HK: native chain, light chain, cleaved light chain of HK respectively.
(TIF)

S2 Fig Correlation between HK cleavage and N-HK plasma concentration and susceptibility to experimental conditions. A, B: Scatter plot of N-HK concentration and HK cleavage in samples from males (A) and females (B). Dotted lines materialized the thresholds defined in Fig 3. ●: healthy donor plasma; ○: nC1Inh-AE plasma. C, D: Scatter plot of N-HK concentration (C) and HK cleavage (D) determined on (n = 32) samples according to the protocol described in the Material and Methods section or using an alternative protocol, optimized to detect faint LC-HK and cLC-HK bands. The alternative protocol includes: a 1-μL plasma volume load onto gels, saturation of nitrocellulose membrane by 1% bovine serum albumin, immunoblotting carried out using anti-HK light chain antibody at a 1/10,000 dilution, and chemi-luminescence performed using ECL Amersham (Arlington Heights, IL USA). HK: high-molecular-weight kininogen; N-HK: native chain of HK.
(TIF)

S3 Fig N-HK concentration and HK cleavage in FXII-HAE and in others nC1Inh-AE patients. No statistical differences were noted but the number of samples (n = 8 and n = 64, respectively) are not sufficient to draw formal conclusions.
(TIF)

S1 File Listing of analysed samples. In this Excel file, the “list” sheet recapitulates all processed samples, in a roughly chronological order. In the early phase (lower “key” numbers) of this study more unrelated samples were analyzed than in the final stages. “Rémi’s” data were acquired with the alternative protocol described in S2 Fig, “Pauline’s” data with the main protocol. Samples analyzed by both protocols are compared in the “robustness” sheet. The inter assay variability was analyzed in the “reproducibility “sheet, with corresponding raw data in the “358–360”sheets. Quantification details from “Pauline’s” data can be found in sheet SM, SF, PM and PF, using the “key” identifier. Columns J-N of the “list” sheet were used for the inclusion/exclusion processing, as described in Material and Methods section, with the following precision: FXII mutation carriers were included in the nC1inh-AE cohort if they were affected (n = 8), healthy carriers were not included. The “final cohort” categorization was used to report data in the “included samples” sheet, and to proceed to statistical analysis.
(XLSX)

S2 File Raw western blot images. Western blot data are sorted by name of Figure, Panel and Patient. Files provided are.scn for the raw data from Biorad software,.tif for the raw image (although gray scale coding might impair viewing by some software);.jpg for the displayed unmodified image;.pdf for the quantification report. For all blots, lanes1-4 are the 20,40, 80 and 110 ng load standard, lane 5 is the ladder (cropped in Fig.), lanes 6–10 are the samples. Samples are not cropped in Figs 1 and 2. Fig 4 patients: O1-2 are in lanes7-10 of the indicated gel, O3-4 in lanes 6–9, O5-6 in lanes 7–10. Fig 5 patients: W6 is in lanes 8–9, M6 in lanes 9–10, L-Cat in lanes 6–8, N-Cat patients in lanes 6–8.
(7Z) Click here for additional data file.

## Abbreviations

AE: angioedema
ACEi-AE: AE linked to angiotensin-converting enzyme inhibitor treatment
BK: bradykinin
C1Inh: C1Inhibitor
C1Inh-AE: angioedema with C1Inh deficiency
FXIIa: activated Factor XII
FXII-AE: AE with mutation of factor XII
HK: high-molecular-weight kininogen
KK: kallikrein
LC-HK and cLC-HK: light chain and cleaved light chain of HK, respectively
nC1Inh-AE: angioedema with normal C1Inh
OP: oral oestrogen/progestin combination
N-HK: native, single-chain HK
ROC curve: receiver operating characteristic curve
I-AE: idiopathic angioedema with unknown aetiology

## References

[1] Cicardi M, Aberer W, Banerji A, Bas M, Bernstein JA, Bork K, et al Classification, diagnosis, and approach to treatment for angioedema: consensus report from the Hereditary Angioedema International Working Group. Allergy. 2014;69: 602–616. 10.1111/all.12380 24673465

[2] Dessart P, Defendi F, Humeau H, Nicolie B, Sarre M-E, Charignon D, et al Distinct conditions support a novel classification for bradykinin-mediated angio-oedema. Dermatology (Basel). 2015;230: 324–331. 10.1159/000371814 25720836

[3] Zuraw BL. Clinical practice. Hereditary angioedema. N Engl J Med. 2008;359: 1027–1036. 10.1056/NEJMcp0803977 18768946

[4] Zuraw BL, Bork K, Binkley KE, Banerji A, Christiansen SC, Castaldo A, et al Hereditary angioedema with normal C1 inhibitor function: consensus of an international expert panel. Allergy Asthma Proc. 2012;33 Suppl 1: S145–156. 10.2500/aap.2012.33.3627 23394603

[5] Walford HH, Zuraw BL. Current update on cellular and molecular mechanisms of hereditary angioedema. Ann Allergy Asthma Immunol. 2014;112: 413–418. 10.1016/j.anai.2013.12.023 24484972

[6] Cugno M, Zanichelli A, Foieni F, Caccia S, Cicardi M. C1-inhibitor deficiency and angioedema: molecular mechanisms and clinical progress. Trends Mol Med. 2009;15: 69–78. 10.1016/j.molmed.2008.12.001 19162547

[7] Nussberger J, Cugno M, Amstutz C, Cicardi M, Pellacani A, Agostoni A. Plasma bradykinin in angio-oedema. Lancet. 1998;351: 1693–1697. 10.1016/S0140-6736(97)09137-X 9734886

[8] Fields T, Ghebrehiwet B, Kaplan AP. Kinin formation in hereditary angioedema plasma: evidence against kinin derivation from C2 and in support of “spontaneous” formation of bradykinin. Journal of Allergy and Clinical Immunology. 1983;72: 54–60. 10.1016/0091-6749(83)90052-0 6222104

[9] Cichon S, Martin L, Hennies HC, Müller F, Van Driessche K, Karpushova A, et al Increased activity of coagulation factor XII (Hageman factor) causes hereditary angioedema type III. Am J Hum Genet. 2006;79: 1098–1104. 10.1086/509899 17186468 PMC1698720

[10] Zuraw BL, Bork K, Binkley KE, Banerji A, Christiansen SC, Castaldo A, et al Hereditary angioedema with normal C1 inhibitor function: consensus of an international expert panel. Allergy Asthma Proc. 2012;33 Suppl 1: S145–156. 10.2500/aap.2012.33.3627 23394603

[11] Kiss N, Barabás E, Várnai K, Halász A, Varga LÁ, Prohászka Z, et al Novel duplication in the F12 gene in a patient with recurrent angioedema. Clin Immunol. 2013;149: 142–145. 10.1016/j.clim.2013.08.001 23994767

[12] Björkqvist J, de Maat S, Lewandrowski U, Di Gennaro A, Oschatz C, Schönig K, et al Defective glycosylation of coagulation factor XII underlies hereditary angioedema type III. J Clin Invest. 2015;125: 3132–3146. 10.1172/JCI77139 26193639 PMC4563738

[13] Charignon D, Ghannam A, Defendi F, Ponard D, Monnier N, López Trascasa M, et al Hereditary angioedema with F12 mutation: factors modifying the clinical phenotype. Allergy. 2014;69: 1659–1665. 10.1111/all.12515 25134986

[14] Gómez-Traseira C, López-Lera A, Drouet C, López-Trascasa M, Pérez-Fernández E, Favier B, et al Hereditary angioedema caused by the p.Thr309Lys mutation in the F12 gene: a multifactorial disease. J Allergy Clin Immunol. 2013;132: 986–989. 10.1016/j.jaci.2013.04.032 23849223

[15] Binkley KE, Davis AE. Estrogen-dependent inherited angioedema. Transfus Apher Sci. 2003;29: 215–219. 10.1016/j.transci.2003.08.002 14572812

[16] Hentges F, Hilger C, Kohnen M, Gilson G. Angioedema and estrogen-dependent angioedema with activation of the contact system. J Allergy Clin Immunol. 2009;123: 262–264. 10.1016/j.jaci.2008.10.056 19130939

[17] Vitrat-Hincky V, Gompel A, Dumestre-Perard C, Boccon-Gibod I, Drouet C, Cesbron JY, et al Type III hereditary angio-oedema: clinical and biological features in a French cohort. Allergy. 2010;65: 1331–1336. 10.1111/j.1398-9995.2010.02368.x 20384613

[18] Blais C, Marceau F, Rouleau JL, Adam A. The kallikrein-kininogen-kinin system: lessons from the quantification of endogenous kinins. Peptides. 2000;21: 1903–1940. 10.1016/S0196-9781(00)00348-X 11150653

[19] Rasmussen ER, Valente de Freitas P, Bygum A. Urticaria and Prodromal Symptoms Including Erythema Marginatum in Danish Patients with Hereditary Angioedema. Acta Derm Venereol. 2015; 10.2340/00015555-2233 26336842

[20] Kaplan AP, Greaves MW. Angioedema. J Am Acad Dermatol. 2005;53: 373–388; quiz 389–392. 10.1016/j.jaad.2004.09.032 16112343

[21] Mandle RJ, Colman RW, Kaplan AP. Identification of prekallikrein and high-molecular-weight kininogen as a complex in human plasma. Proceedings of the National Academy of Sciences. 1976;73: 4179–4183. 10.1073/pnas.73.11.4179 1069308 PMC431375

[22] Tankersley DL, Finlayson JS. Kinetics of activation and autoactivation of human factor XII. Biochemistry. 1984;23: 273–279. 10.1021/bi00297a016 6607744

[23] Davis AE 3rd, Lu F, Mejia P. C1 inhibitor, a multi-functional serine protease inhibitor. Thromb Haemost. 2010;104: 886–893. 10.1160/TH10-01-0073 20806108

[24] Cyr M, Lepage Y, Blais C, Gervais N, Cugno M, Rouleau JL, et al Bradykinin and des-Arg(9)-bradykinin metabolic pathways and kinetics of activation of human plasma. Am J Physiol Heart Circ Physiol. 2001;281: H275–283. 11406494 10.1152/ajpheart.2001.281.1.H275

[25] Ghannam A, Sellier P, Defendi F, Favier B, Charignon D, López-Lera A, et al C1 inhibitor function using contact-phase proteases as target: evaluation of an innovative assay. Allergy. 2015;70: 1103–1111. 10.1111/all.12657 26010015

[26] Defendi F, Charignon D, Ghannam A, Baroso R, Csopaki F, Allegret-Cadet M, et al Enzymatic assays for the diagnosis of bradykinin-dependent angioedema. PLoS ONE. 2013;8: e70140 10.1371/journal.pone.0070140 23940538 PMC3734293

[27] Joseph K, Bains S, Tholanikunnel BG, Bygum A, Aabom A, Koch C, et al A novel assay to diagnose hereditary angioedema utilizing inhibition of bradykinin-forming enzymes. Allergy. 2015;70: 115–119. 10.1111/all.12520 25186184

[28] Drouet C, Désormeaux A, Robillard J, Ponard D, Bouillet L, Martin L, et al Metallopeptidase activities in hereditary angioedema: effect of androgen prophylaxis on plasma aminopeptidase P. J Allergy Clin Immunol. 2008;121: 429–433. 10.1016/j.jaci.2007.10.048 18158172 PMC4126900

[29] Hofman Z, Relan A, Zeeleeder S, Drouet C, Zuraw B, Hack E. Angioedema attacks of hereditary angioedema: Local manifestations of a systemic activation process. The Journal of Allergy and Clinical Immunology. accepted; 10.1016/j.jaci.2016.02.041 27246526

[30] Robillard J, Gauvin F, Molinaro G, Leduc L, Adam A, Rivard GE. The syndrome of amniotic fluid embolism: A potential contribution of bradykinin. Am J Obstet Gynecol. 2005;193: 1508–1512. 10.1016/j.ajog.2005.03.022 16202747

[31] Colman RW, Schmaier AH. Contact system: a vascular biology modulator with anticoagulant, profibrinolytic, antiadhesive, and proinflammatory attributes. Blood. 1997;90: 3819–3843. 9354649

[32] Giard C, Nicolie B, Drouet M, Lefebvre-Lacoeuille C, Le Sellin J, Bonneau J-C, et al Angio-oedema induced by oestrogen contraceptives is mediated by bradykinin and is frequently associated with urticaria. Dermatology. 2012;225: 62–69. 10.1159/000340029 22922353

[33] Kahn R, Herwald H, Müller-Esterl W, Schmitt R, Sjögren A-C, Truedsson L, et al Contact-system activation in children with vasculitis. Lancet. 2002;360: 535–541. 10.1016/S0140-6736(02)09743-X 12241658

[34] Drouet C, Alibeu C, Ponard D, Arlaud GJ, Colomb MG. A sensitive method to assay blood complement C1- inhibitor activity. Clin Chim Acta. 1988;174: 121–130. 3260154 10.1016/0009-8981(88)90379-8

[35] McNeil BJ, Hanley JA. Statistical approaches to the analysis of receiver operating characteristic (ROC) curves. Med Decis Making. 1984;4: 137–150. 10.1177/0272989X8400400203 6472062

[36] Scott CF, Silver LD, Schapira M, Colman RW. Cleavage of human high molecular weight kininogen markedly enhances its coagulant activity. Evidence that this molecule exists as a procofactor. J Clin Invest. 1984;73: 954–962. 10.1172/JCI111319 6561202 PMC425106

[37] Thompson RE, Mandle R, Kaplan A. Characterization of human high molecular weight kininogen. Procoagulant activity associated with the light chain of kinin-free high molecular weight kininogen. The Journal of experimental medicine. 1978;147: 488–499. 10.1084/jem.147.2.488 75240 PMC2184499

[38] Suffritti C, Zanichelli A, Maggioni L, Bonanni E, Cugno M, Cicardi M. High-molecular-weight kininogen cleavage correlates with disease states in the bradykinin-mediated angioedema due to hereditary C1-inhibitor deficiency. Clin Exp Allergy. 2014;44: 1503–1514. 10.1111/cea.12293 24552232

[39] Nussberger J, Cugno M, Cicardi M. Bradykinin-mediated angioedema. N Engl J Med. 2002;347: 621–622. 10.1056/NEJM200208223470820 12192030

[40] Sala-Cunill A, Björkqvist J, Senter R, Guilarte M, Cardona V, Labrador M, et al Plasma contact system activation drives anaphylaxis in severe mast cell-mediated allergic reactions. J Allergy Clin Immunol. 2015;135: 1031–1043.e6. 10.1016/j.jaci.2014.07.057 25240785

[41] Kaplan AP, Ghebrehiwet B. The plasma bradykinin-forming pathways and its interrelationships with complement. Mol Immunol. 2010;47: 2361–2169. 10.1016/j.molimm.2010.05.010 20580091

[42] Adam A, Albert A, Calay G, Closset J, Damas J, Franchimont P. Human kininogens of low and high molecular mass: quantification by radioimmunoassay and determination of reference values. Clin Chem. 1985;31: 423–426. 3971563

[43] Zamolodchikov D, Chen Z-L, Conti BA, Renné T, Strickland S. Activation of the factor XII-driven contact system in Alzheimer’s disease patient and mouse model plasma. Proc Natl Acad Sci USA. 2015;112: 4068–4073. 10.1073/pnas.1423764112 25775543 PMC4386355

[44] Cugno M, Cicardi M, Bottasso B, Coppola R, Paonessa R, Mannucci PM, et al Activation of the coagulation cascade in C1-inhibitor deficiencies. Blood. 1997;89: 3213–3218. 9129025

[45] Hui SL, Walter SD. Estimating the error rates of diagnostic tests. Biometrics. 1980;36: 167–171. 10.2307/2530508 7370371

[46] Drouet C, Khoy K, Masson D, Bardy B, Giannoli C, Dubois V. [The immunological conflict in the transfusion-related acute lung injury or TRALI]. Transfus Clin Biol. 2011;18: 224–229. 10.1016/j.tracli.2011.02.010 21470890

