# Supplementary figures and images for: Kininogen Cleavage Assay: Diagnostic Assistance for Kinin-Mediated Angioedema Conditions
Source: PLoS One. 2016 Sep 29;11(9):e0163958. doi: 10.1371/journal.pone.0163958 (PMC5042432; doi:10.1371/journal.pone.0163958)

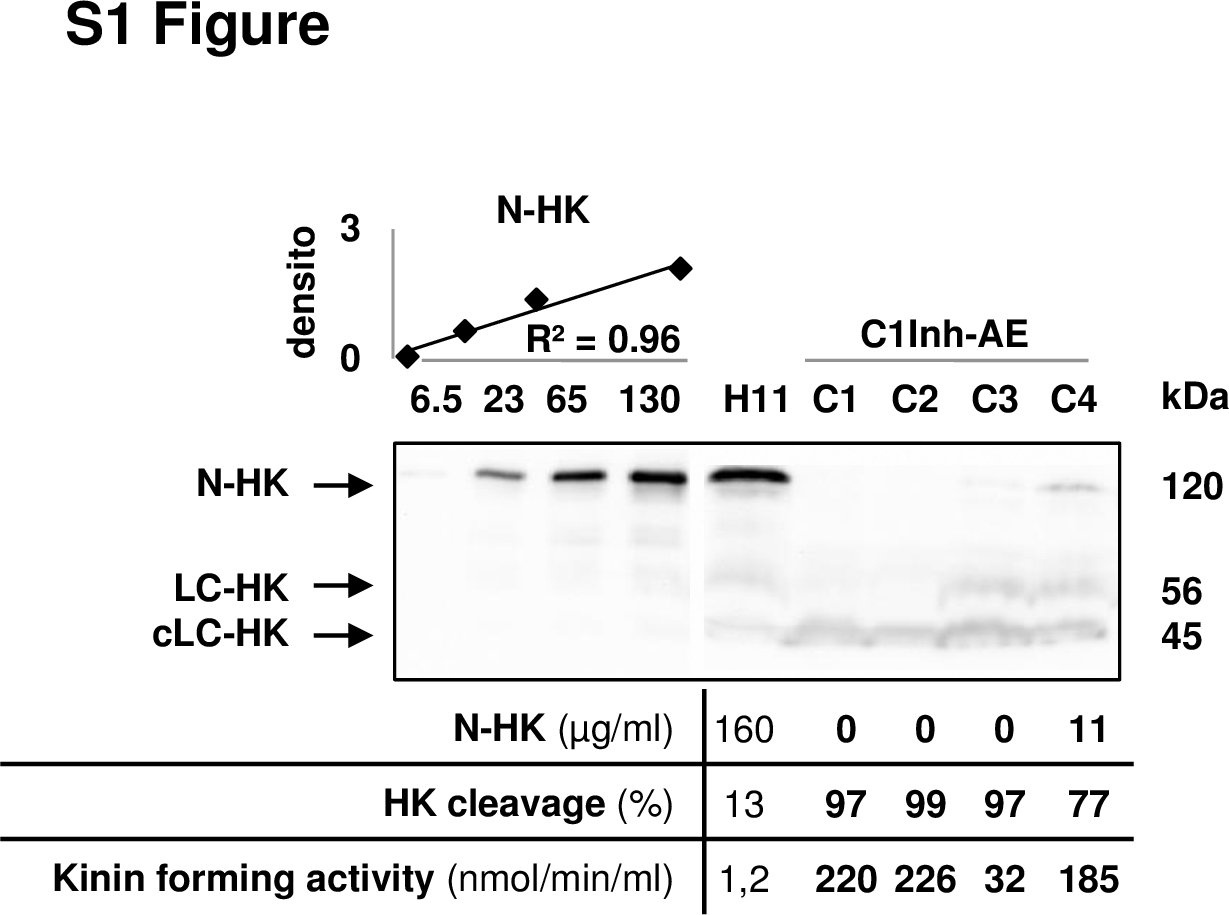

Supplement: S1 Fig — High-molecular-weight kininogen (HK) molecular pattern of plasma samples from one control (H11) and C1Inh-AE patients (n = 4; C1–C4), analysed as in Fig 1. N-HK concentrations were evaluated by the displayed linear regression, kinin-forming activity as in [26], and pathological values were labelled in bold fonts. densito: densitometry in 107 arbitrary units; C1Inh-AE; angioedema with C1Inh deficiency; N-HK, LC-HK, cLC-HK: native chain, light chain, cleaved light chain of HK respectively. (TIF) [file pone.0163958.s001.tif]

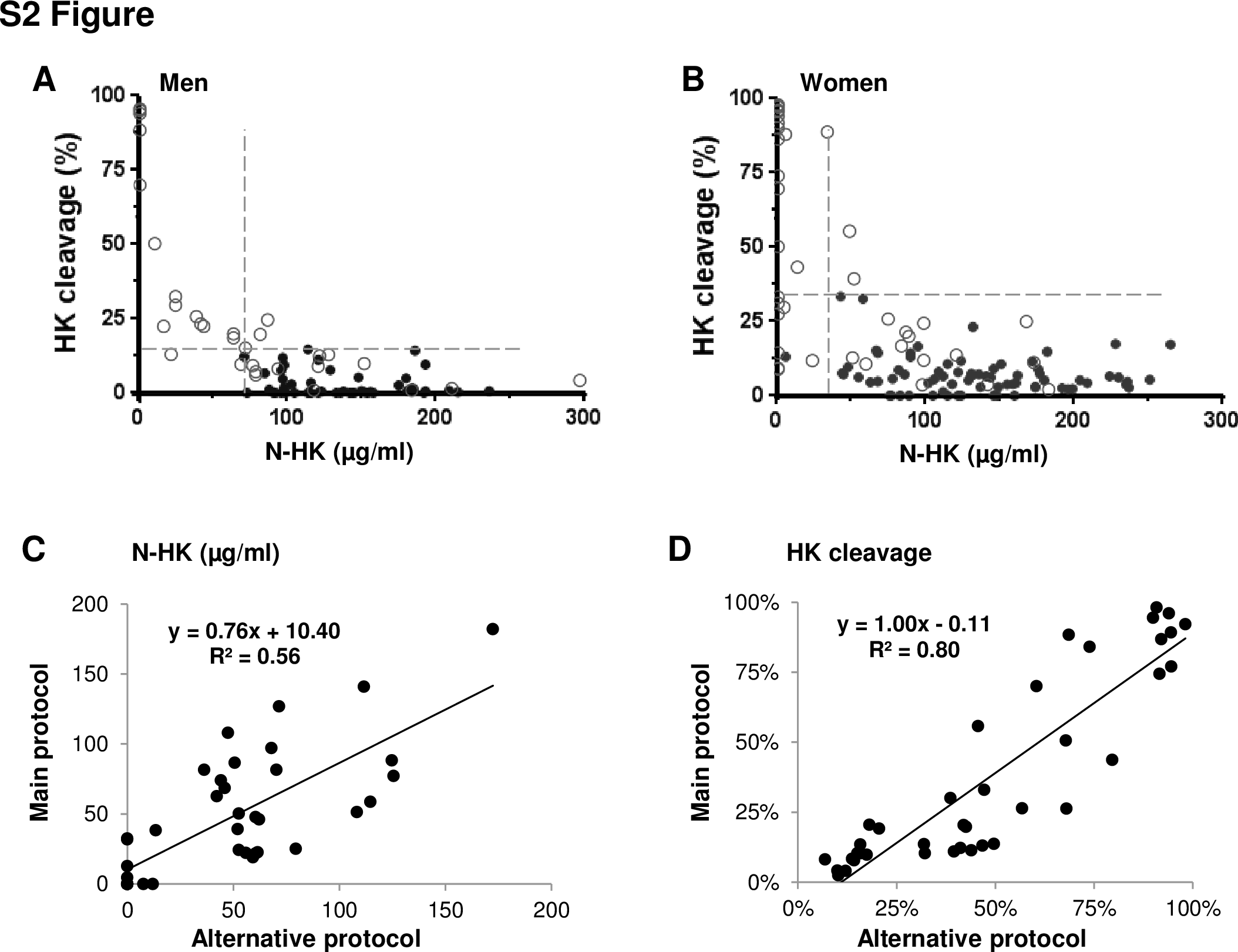

Supplement: S2 Fig — A, B: Scatter plot of N-HK concentration and HK cleavage in samples from males (A) and females (B). Dotted lines materialized the thresholds defined in Fig 3. ●: healthy donor plasma; ○: nC1Inh-AE plasma. C, D: Scatter plot of N-HK concentration (C) and HK cleavage (D) determined on (n = 32) samples according to the protocol described in the Material and Methods section or using an alternative protocol, optimized to detect faint LC-HK and cLC-HK bands. The alternative protocol includes: a 1-μL plasma volume load onto gels, saturation of nitrocellulose membrane by 1% bovine serum albumin, immunoblotting carried out using anti-HK light chain antibody at a 1/10,000 dilution, and chemi-luminescence performed using ECL Amersham (Arlington Heights, IL USA). HK: high-molecular-weight kininogen; N-HK: native chain of HK. (TIF) [file pone.0163958.s002.tif]

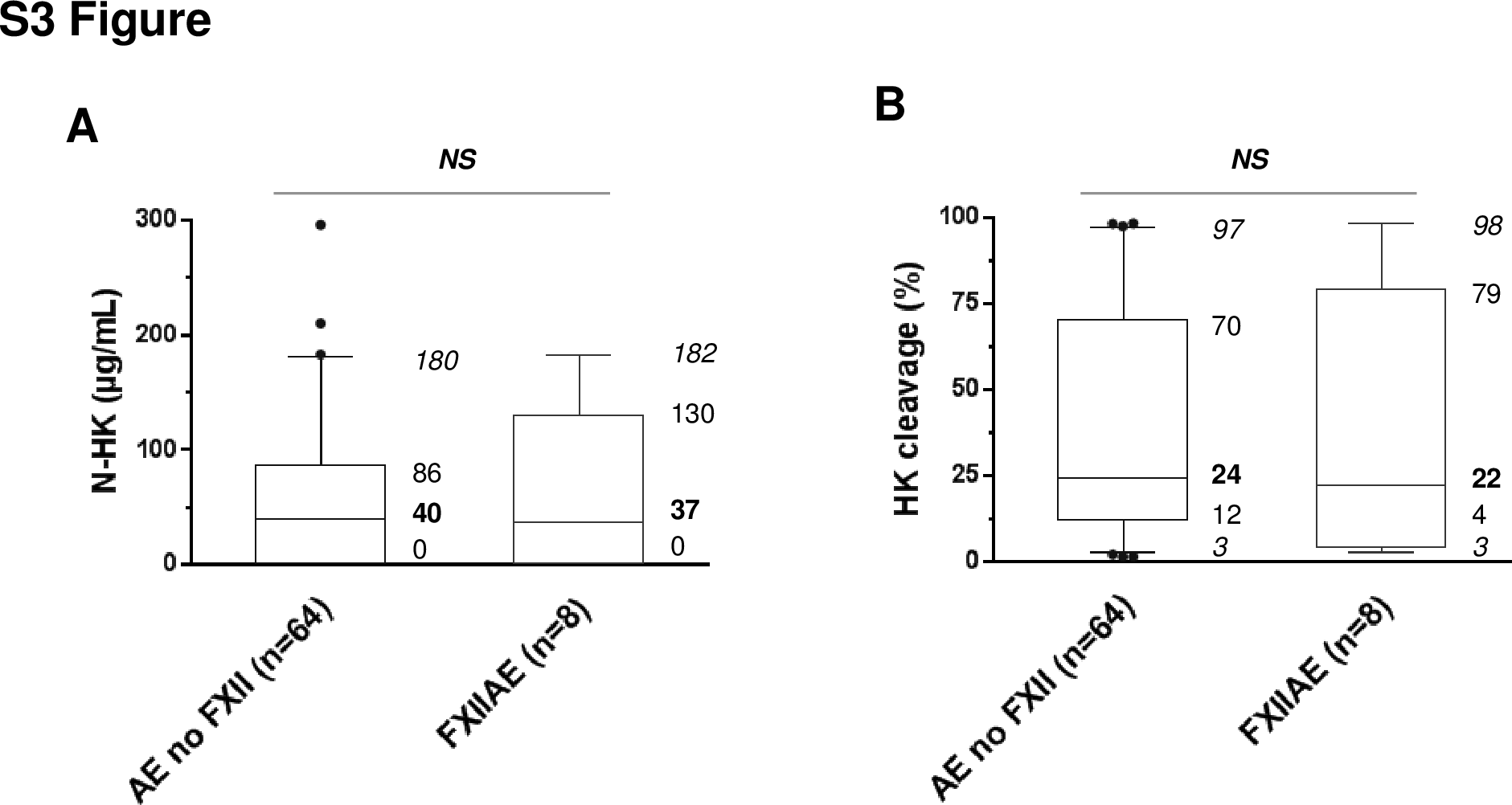

Supplement: S3 Fig — No statistical differences were noted but the number of samples (n = 8 and n = 64, respectively) are not sufficient to draw formal conclusions. (TIF) [file pone.0163958.s003.tif]
